# Supplementary material for: Optimized Spheroid Model of Pancreatic Cancer Demonstrates Influence of Macrophage–T Cell Interaction for Intratumoral T Cell Motility
Source: Cancers (Basel). 2024 Dec 27;17(1):51. doi: 10.3390/cancers17010051 (PMC11718817; doi:10.3390/cancers17010051)
Supplement: Supplementary file 1 [file cancers-17-00051-s001.zip › Suppl. Figures_Spheroid Model_final.pdf]

Supplementary Figure S1

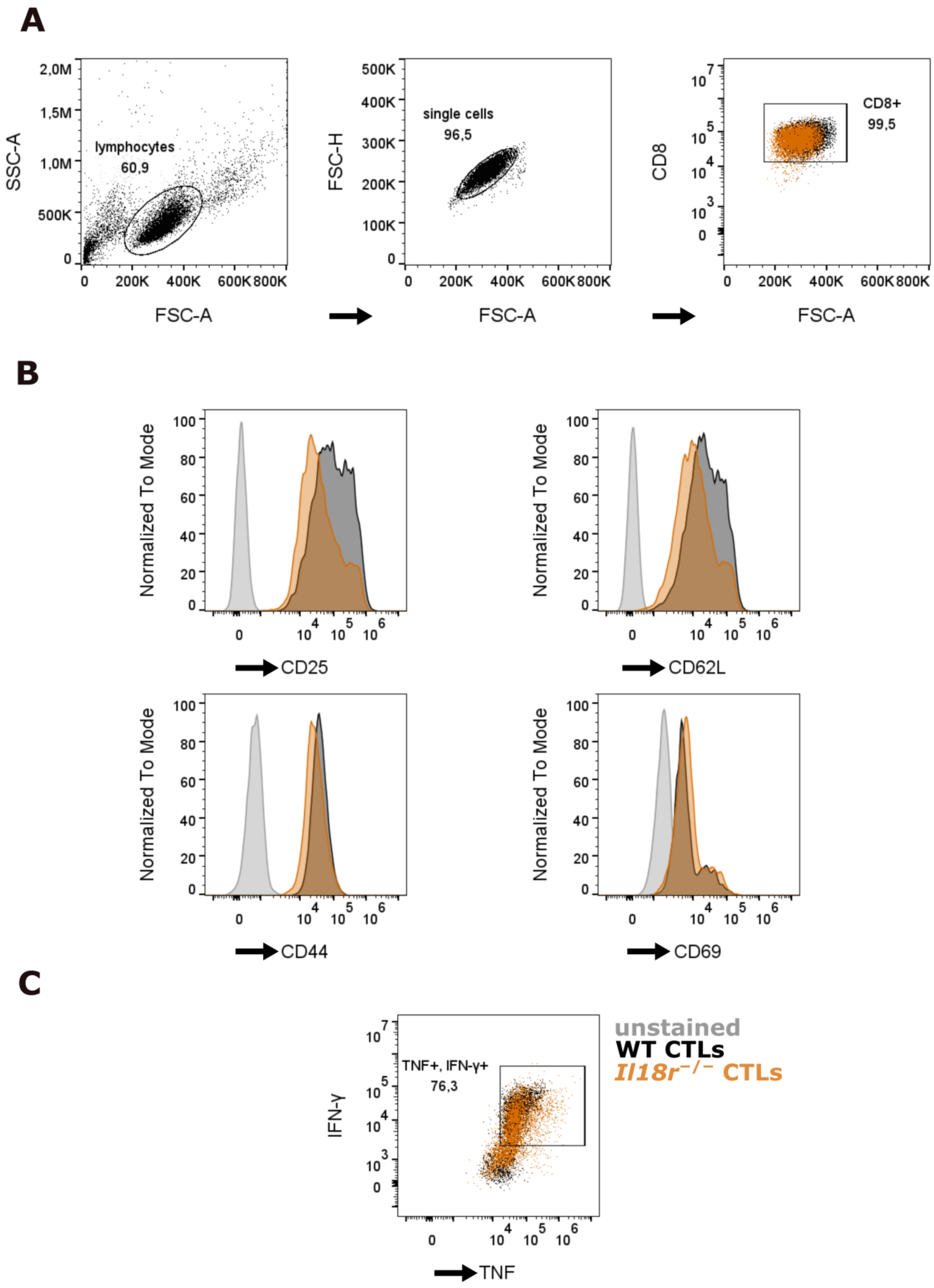

Figure S1: Characterization of WT and *Il18r*$^{-/-}$ CTLs via FACS analysis. (**A**) Common gating strategy identifying viable CD8^{+} T cells for further surface marker and intracellular staining. FACS analysis was performed before adding the cells to the coculture. Cells were first gated for lymphocytes (SSC-A / FSC-A), afterwards gated for single cells (FSC-H / FSC-A) and finally for the expression of CD8. Then, CD8^{+} WT and *Il18r*$^{-/-}$ CTLs were analyzed for surface markers and underwent intracellular staining. (**B**) Expression of surface markers CD44, CD25, CD69 and CD62L from viable CD8^{+} T cells. (**C**) Intracellular staining for IFN-γ and TNF in viable CD8^{+} T cells.

Supplementary Figure S2

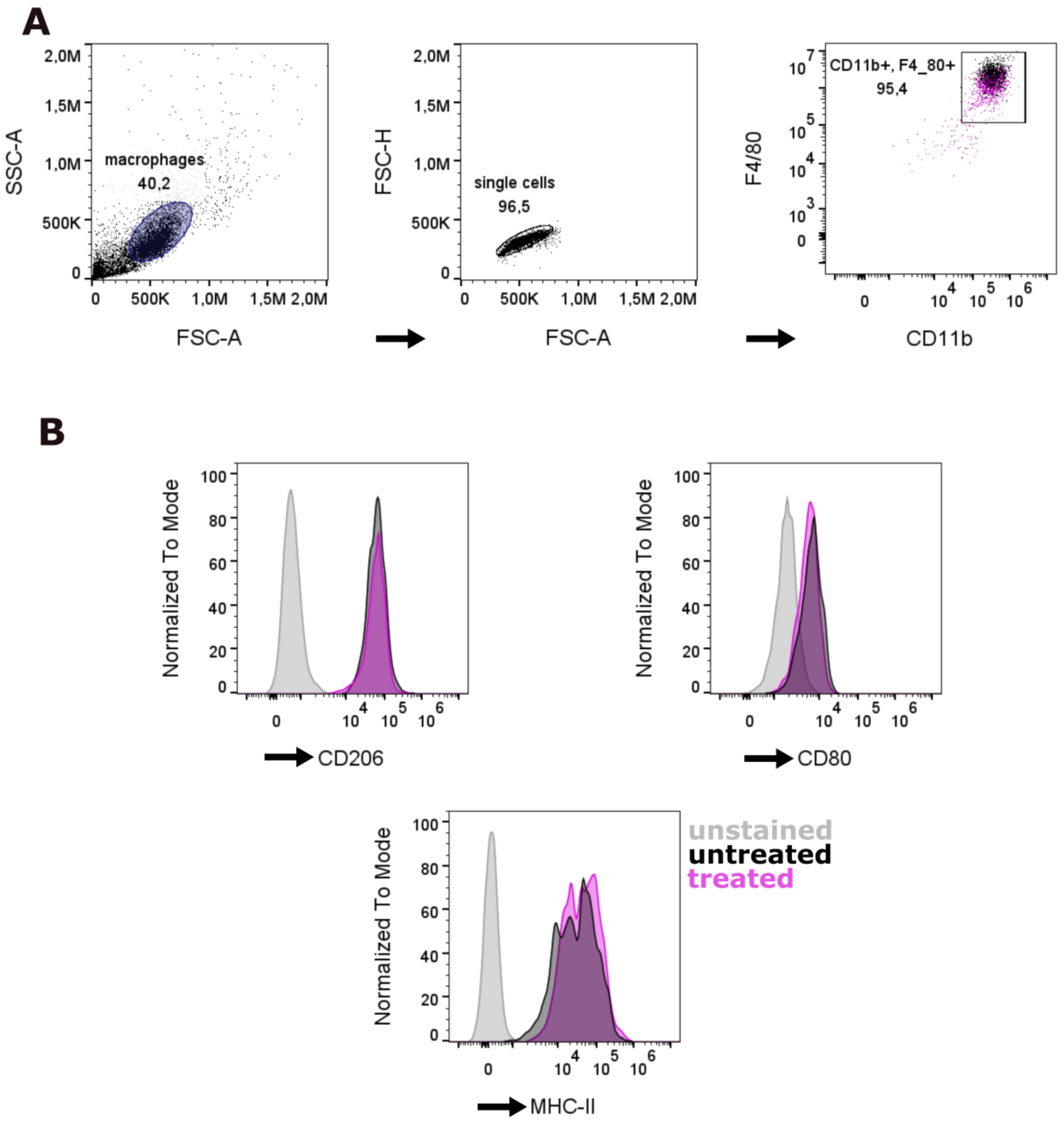

Figure S2: Characterization of macrophages via FACS analysis. (**A**) Gating strategy identifying viable macrophages with or without LPS and nigericin pre-treatment. FACS analysis was performed before adding the cells to the coculture. Cells were first gated for macrophages (SSC-A / FSC-A), then for single cells (FSC-H / FSC-A) and finally for F4/80$^+$ and CD11b$^+$ cells. (**B**) Expression of surface markers CD80, CD206 and MHC-II from viable macrophages.

Supplementary Figure S3

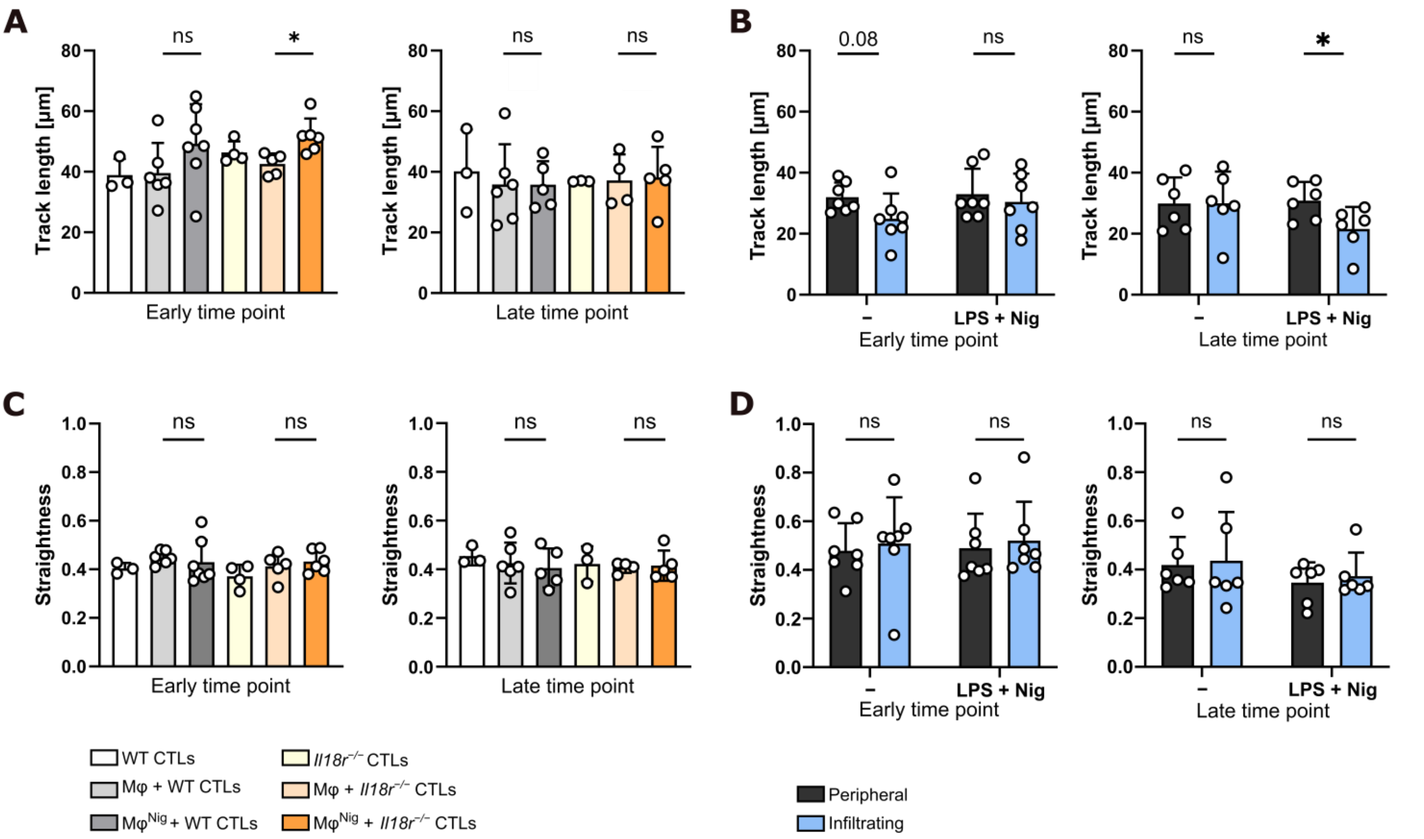

Figure S3: Treatment of macrophages with LPS and nigericin leads to longer but not straighter tracks of CTLs. The migration of spheroid-infiltrating CTLs and macrophages was analyzed through Imaris-based tracking of 3D videos with 30-minute recording time each. (**A**) Track length of infiltrating WT and *Il18r* $^{-/-}$ CD8^{+} CTLs at early and late time points (*n* = 3-7) was determined. (**B**) Similarly, track length of infiltrating and peripheral macrophages at early and late time points (*n* = 6-7) was determined. (**C**) Straightness of WT and *Il18r* $^{-/-}$ CTL motility was calculated in Imaris (*n* = 3-7). Straightness represents directed movement over time. (**D**) Straightness of macrophage motility was calculated (*n* = 6-7). −: no treatment with LPS and nigericin; LPS + Nig: treatment with 200 ng/mL LPS (2 h) and 10 µM nigericin (1 h). Statistical analysis was performed using an unpaired, two-tailed t-test with Welch correction. All graphs are shown as mean ± SD. * p ≤ 0.05.

Supplementary Figure S4

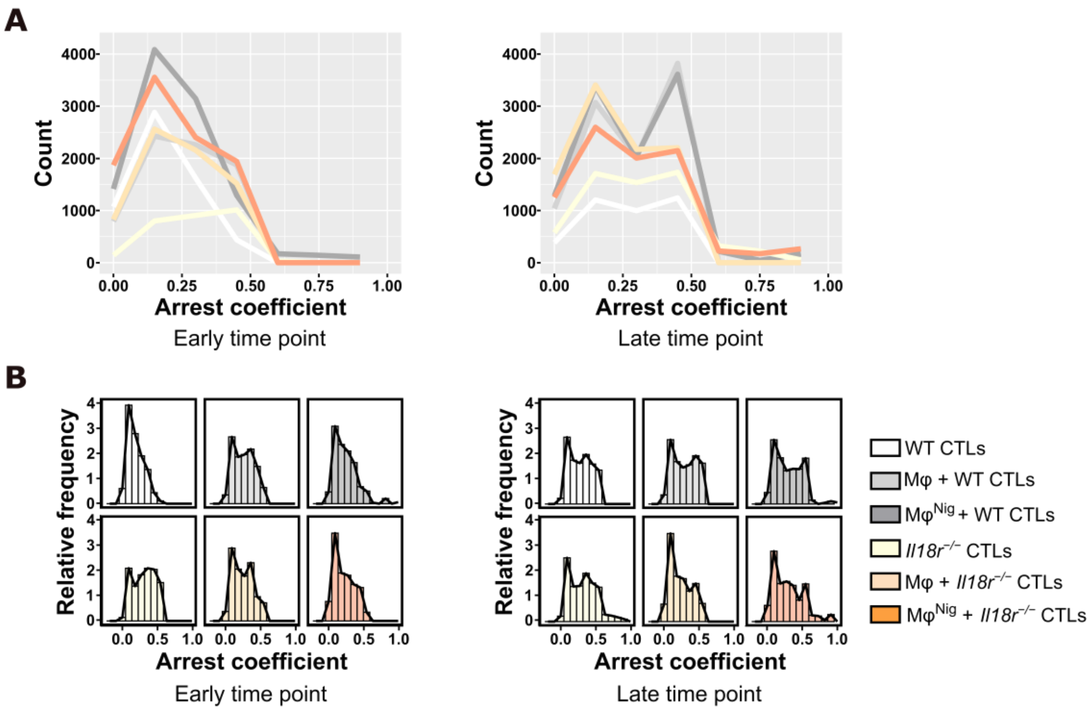

Figure S4: Absolute counts and frequency of arrest states are shifted towards higher arrest coefficients from early to late recording time points. While most CTLs at the early time point (4 h after start of coculture) have low arrest coefficients, CTLs at the late time point (18 h) demonstrate a shift towards higher arrest coefficients and demonstrate an additional maximum at higher arrest coefficients. The migration of spheroid-infiltrating CTLs was analyzed through Imaris-based tracking of 3D videos with a recording time of 30 minutes. Data were processed with RStudio. (**A**) Count of every arrest coefficient from infiltrating WT and *Il18r*$^{-/-}$ CD8^{+} CTLs at the early (4 h) and late (18 h of coculture) time points (*n* = 3-7). (**B**) Relative frequency of arrest coefficients from WT and *Il18r*$^{-/-}$ CD8^{+} CTLs at early and late time points (*n* = 3-7).
